# Supplementary material for: COVA1-18 neutralizing antibody protects against SARS-CoV-2 in three preclinical models
Source: Nat Commun. 2021 Oct 20;12:6097. doi: 10.1038/s41467-021-26354-0 (PMC8528857; doi:10.1038/s41467-021-26354-0)
Supplement: Supplementary file 1 — Supplementary Information [file 41467_2021_26354_MOESM1_ESM.pdf]

COVA1-18 neutralizing antibody protects against SARS-CoV-2 in three preclinical models

Supplementary information

Supplementary figures

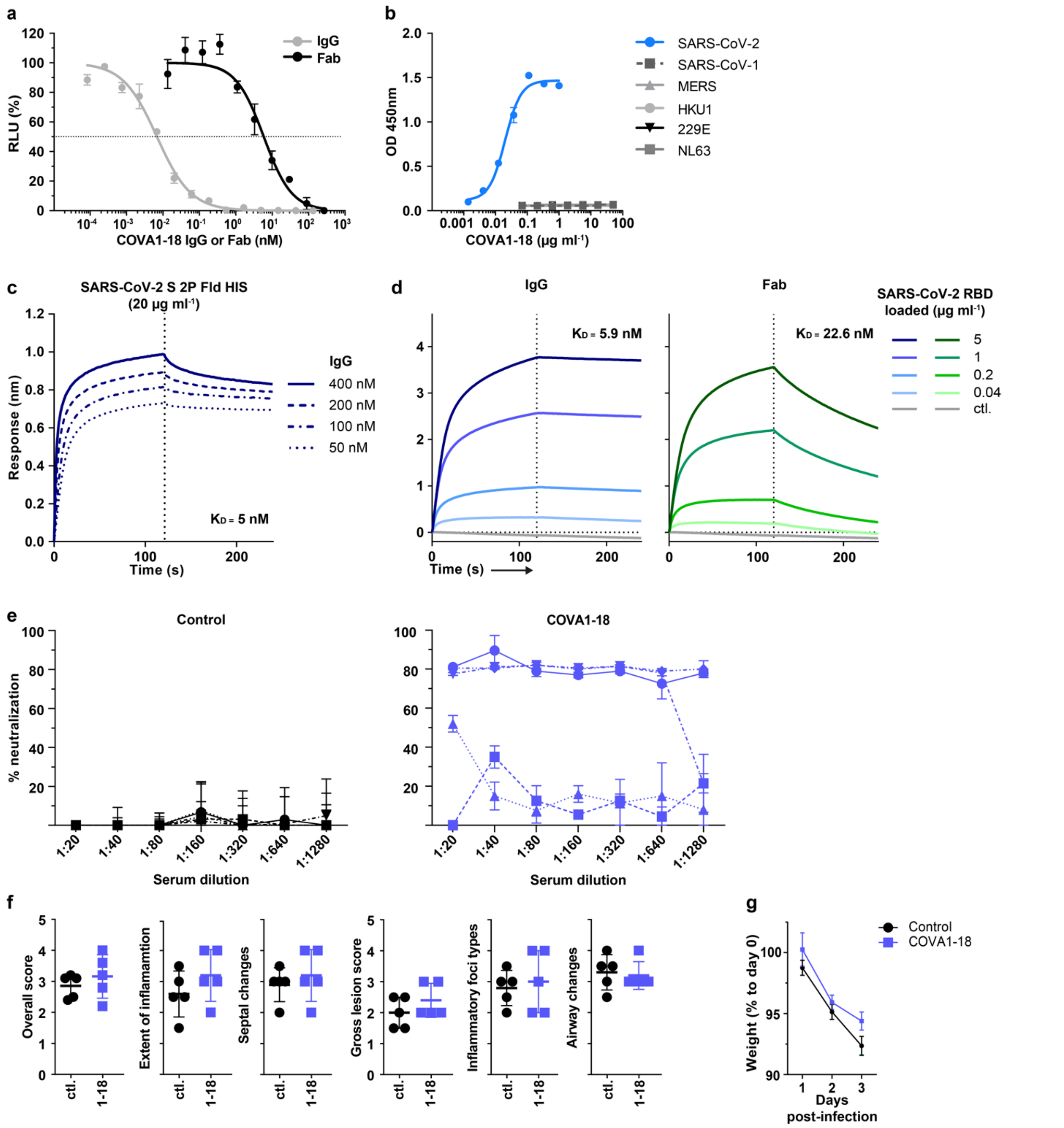

Supplementary Figure 1. COVA1-18 IgG and Fab neutralization, cross-reactivity, binding kinetic and Syrian hamster serum neutralization. (**a**) IgG (grey) and Fab (black) pseudotype particle neutralization curves for COVA1-18 (means ± SEMs of 3 replicates). Representative of n ≥ 4 independent experiments. (**b**) Antigen specificity of COVA1-18 was assessed by ELISA against the soluble S protein derived from different human coronaviruses (means ± SEMs). Representative of n ≥ 2 independent experiments. (**c**) Biolayer Interferometry (BLI) sensorgrams of COVA1-18 binding to immobilized soluble SARS-CoV-2 S protein. Representative of n ≥ 2 independent experiments. (**d**) BLI sensorgrams of COVA1-18 binding to SARS-CoV-2 RBD loaded onto the sensor chip at various concentrations (n = 1). (**e**) Serum neutralization potency at 3 d.p.i. in Syrian hamsters for the control group (left) and COVA1-18 treated group (n = 5 animals per group) (means ± SDs). (**f**) Clinical scores were determined as per Supplementary Table 1 (means ± SDs). (**g**) Animals were weighed daily and weight normalized to day 0 for each animal (means ± SEMs). For (**f**) and (**g**), n = 5 per group. ctl., control; RBD, Receptor-binding domain; RLU, relative luminescence unit; 1-18, COVA1-18.

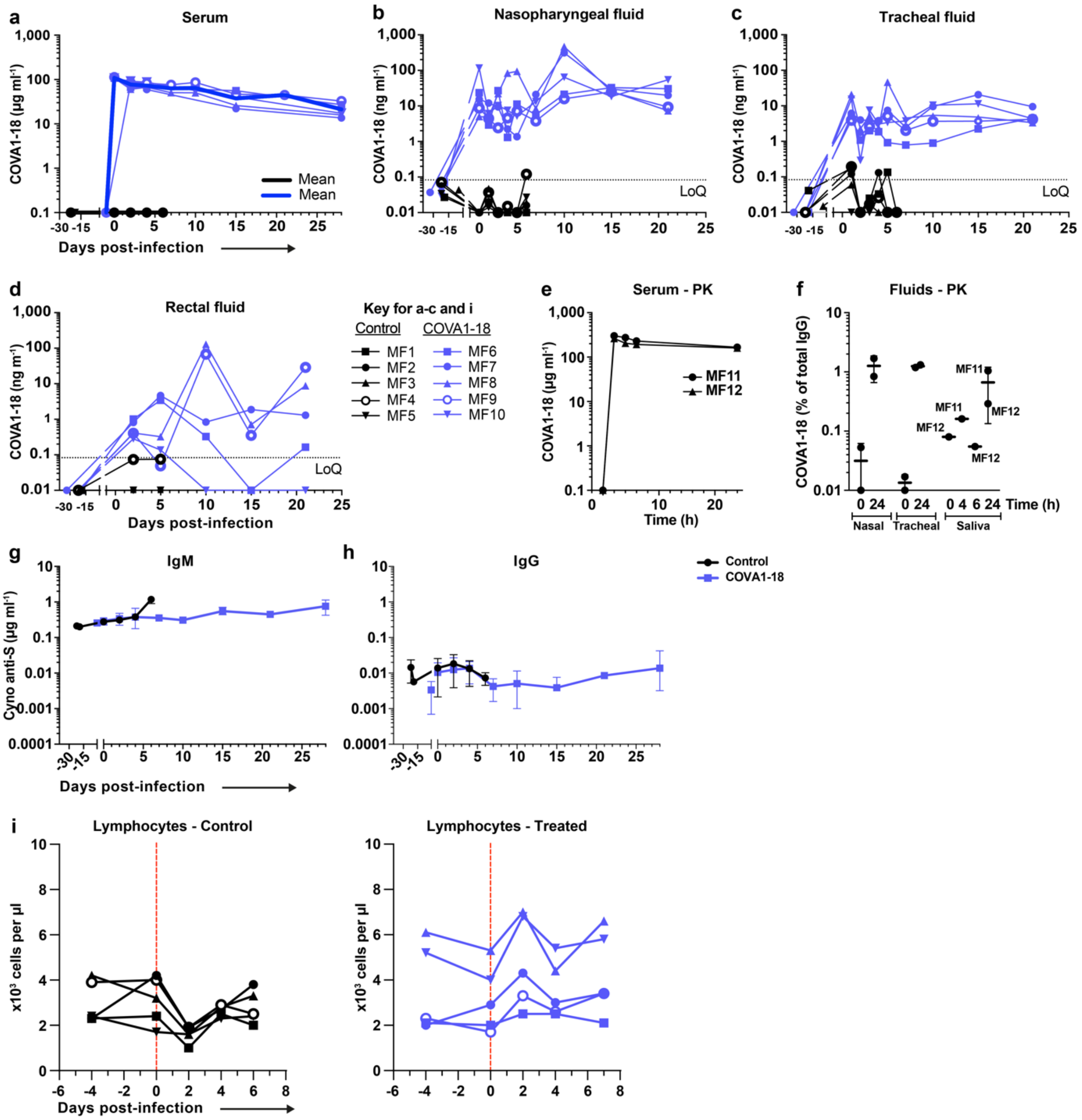

Supplementary Figure 2. Serum and mucosal pharmacokinetics of COVA1-18 in treated macaques, lymphocyte counts and cynomolgus IgM and IgG response. (**a**) Serum COVA1-18 concentration for each animal. The mean COVA-18 concentration for each group is indicated by a thick blue line (treated animals) and a thick black line (control). The COVA1-18 concentrations measured in nasopharyngeal (**b**), tracheal (**c**) and rectal (**d**) fluids by ELISA are reported for each animal in both groups. (**e**) Serum COVA1-18 concentration from two animals injected with 10 mg kg^{-1} of COVA1-18 and sampled at 0, 2, 4, 6 and 24 h for a pharmacokinetic (PK) study. (**f**) COVA1-18 was measured in fluid samples of the PK study animals and normalized to the total cynomolgus IgG content for each sample. Bars represent means (n = 2). (**g**) Cynomolgus anti-S IgM and (**h**) IgG responses (means with range, n = 5 per group). (**i**) Absolute lymphocyte count in the blood of control (left panel) and treated (right panel) animals. LoQ, limit of quantification; MF, Macaca Fascicularis; PK, pharmacokinetic.

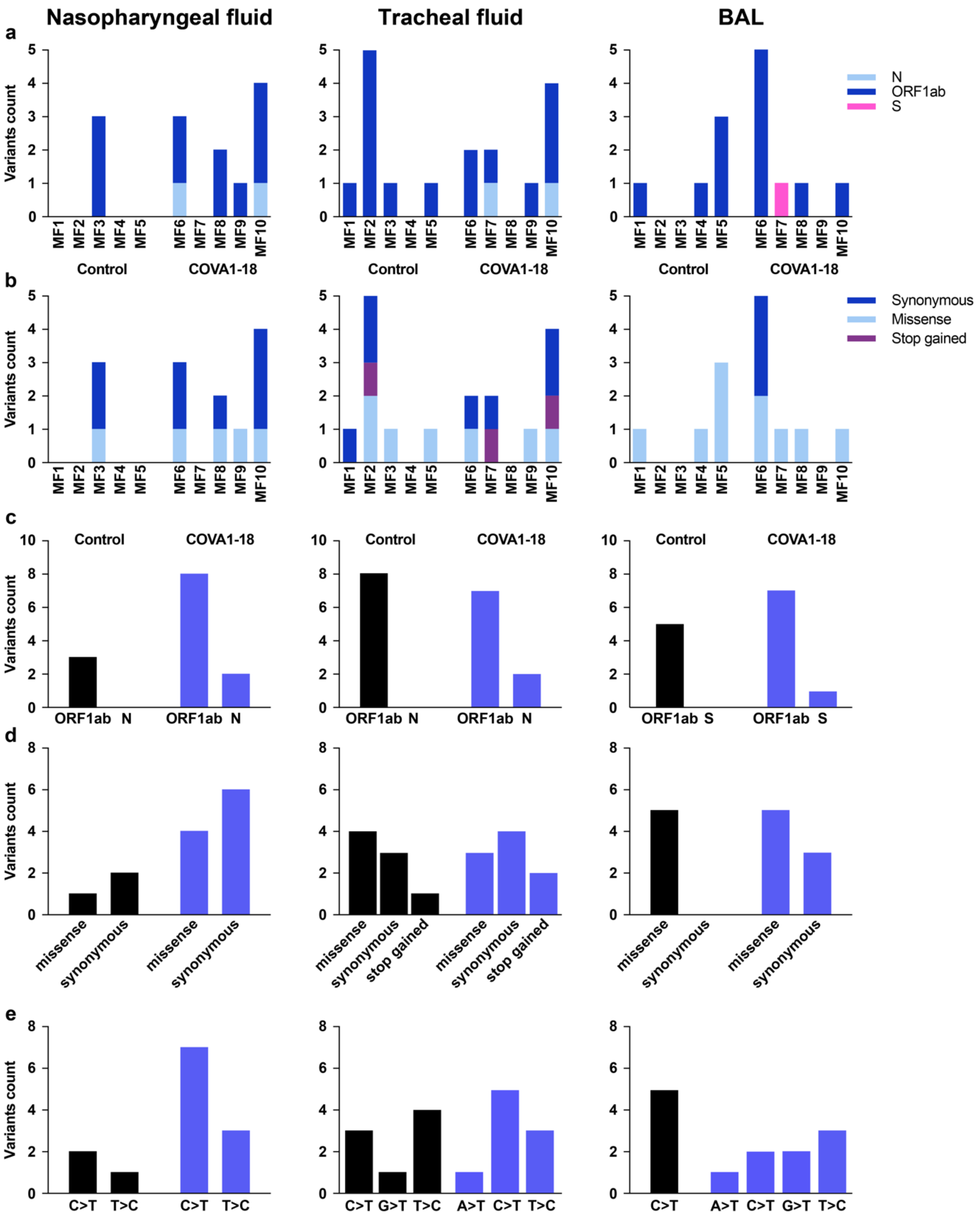

Supplementary Figure 3. Sequences in treated and exposed NHP. Viral population sequences in the nasopharyngeal swabs (left panels), tracheal swabs (middle panels) and BAL fluids (right fluids) at 3 d.p.i. were analyzed by Next Generation Sequencing. (**a**) Variants count detected for each indicated gene for each individual. (**b**) Individual synonymous and missense variants count for the control and treated groups. (**c**) Cumulative variants count for each gene in the control and COVA1-18 treated groups. (**d**) Cumulative synonymous and missense variants count for each group. (**e**) Nucleotide substitution observed by type for both groups. MF, Macaca Fascicularis.

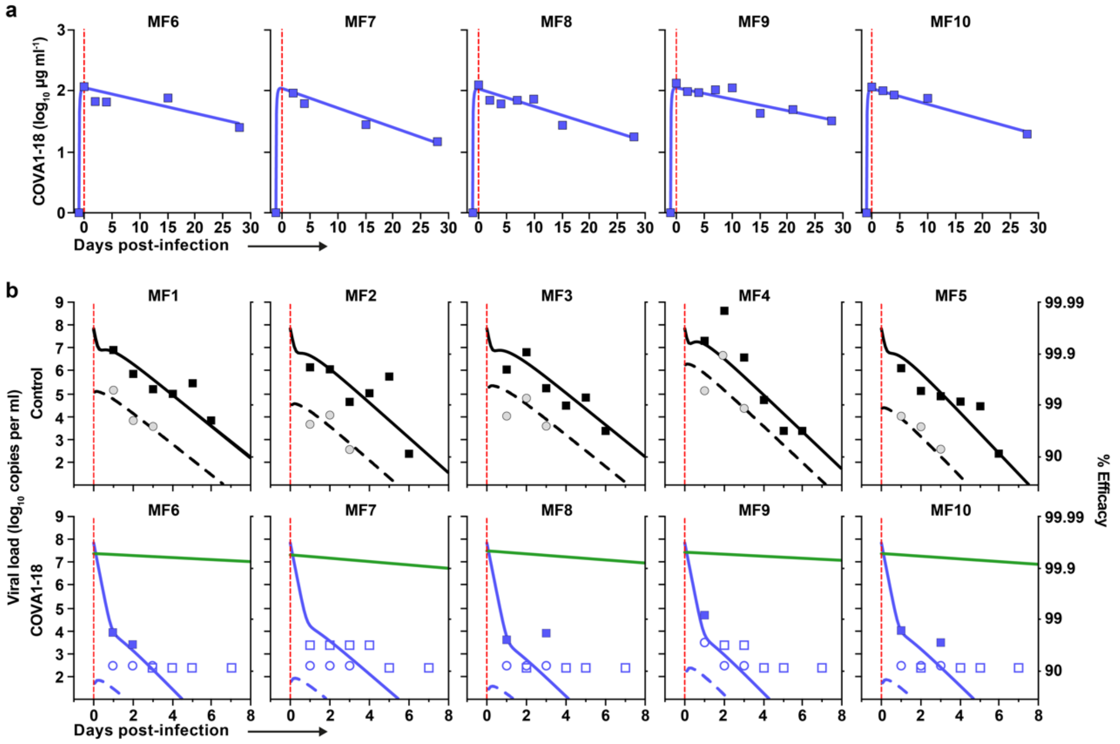

c

		MF6	MF7	MF8	MF9	MF10
Mean efficacy (%)	Nasopharynx	97.34	96.67	97.37	97.50	97.16
	Trachea	99.93	99.91	99.93	99.94	99.93

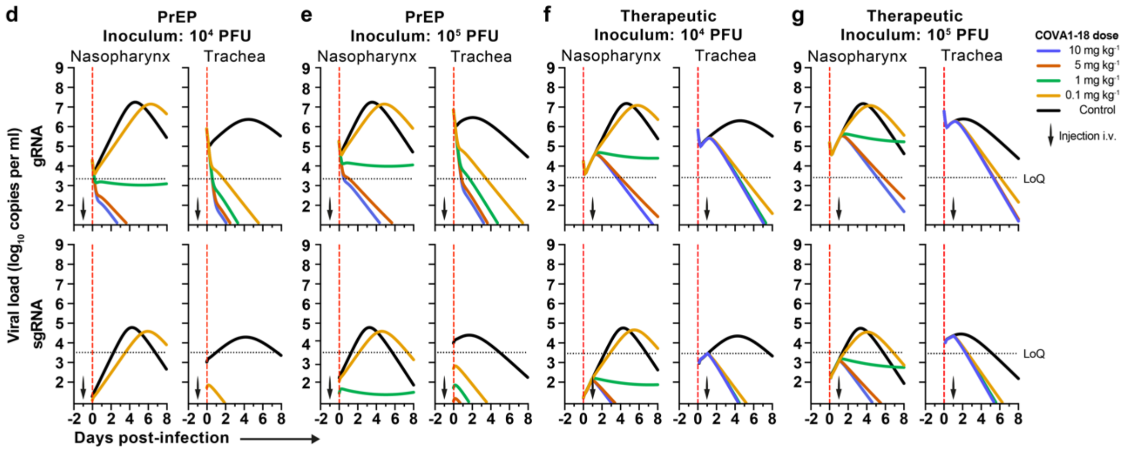

Supplementary Figure 4. Modeling of viral dynamics and treatment efficacy (1/2). (**a**) Individual prediction of the COVA1-18 plasma concentration. (**b**) Individual prediction of the tracheal genomic (g)RNA and subgenomic (sg)RNA in control (top) and treated animals (bottom) with individual efficacy prediction indicated (green line). The dashed red line indicates the time of viral infection. gRNA (squares) and sgRNA (circles) data are indicated as plain (above LoQ) or open (below LoQ). (**c**) Mean individual efficacy of the COVA1-18 for each individual in both compartments (calculated over the first 10 days of administration). (**d-e**) Simulation of the predicted gRNA (top) and sgRNA (bottom) viral loads in the nasopharynx and trachea for a 10^4 and 10^5 PFU challenge dose according to the dose of COVA1-18 given 24 h prior challenge (arrow). (**f-g**) Simulation as in (**d**) with COVA1-18 given 24 h post-infection. Black dotted lines indicate the limit of quantification (LoQ). i.v., intravenous; MF, Macaca Fascicularis; PFU, plaque forming units; PrEP, Pre-Exposure Prophylaxis.

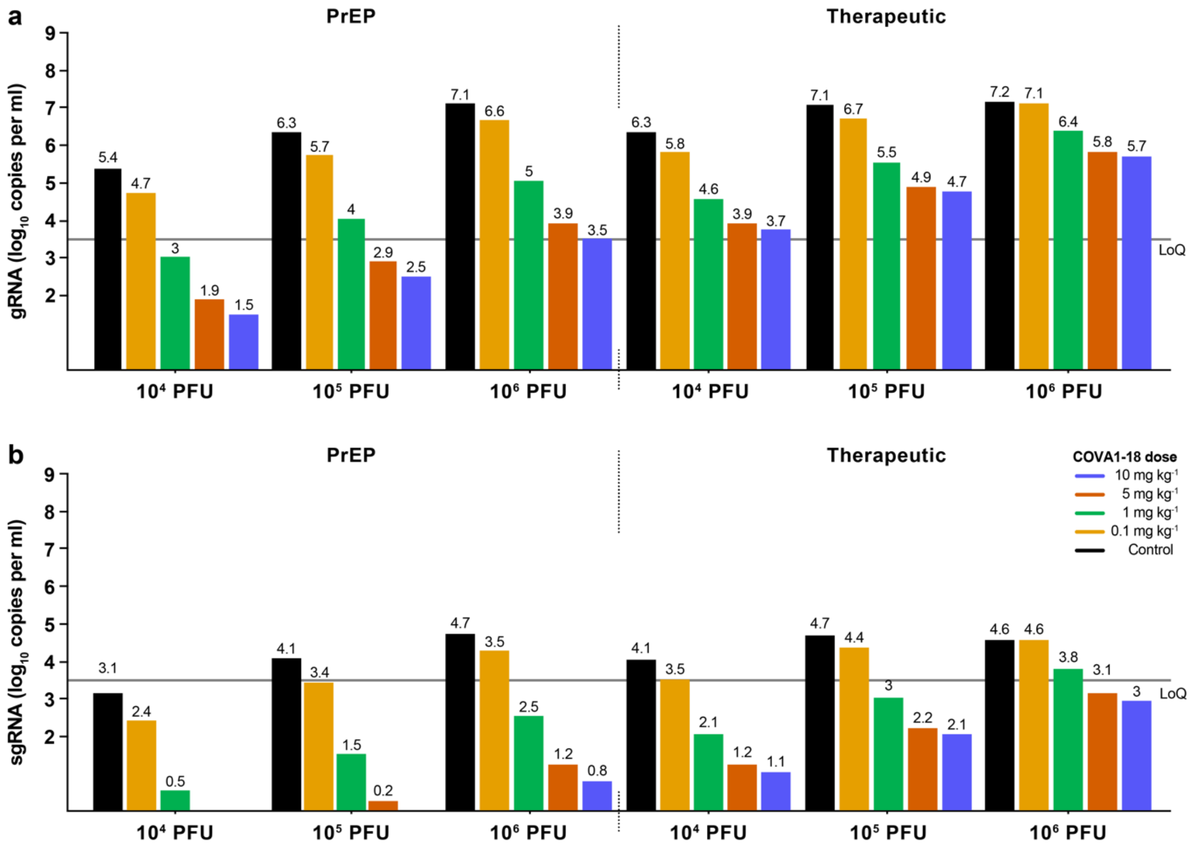

Supplementary Figure 5. Modeling of viral dynamics and treatment efficacy (2/2). Simulation of the predicted genomic (g)RNA (**a**) and subgenomic (sg)RNA (**b**) viral loads in the nasopharynx, according to the dose of COVA1-18 received and the dose of virus received. Left: Pre-Exposure Prophylaxis (PrEP) treatment at -1 d.p.i., viral load measured at 2 d.p.i.; Right: Therapeutic treatment at 1 d.p.i., viral load measured at 3 d.p.i. Black: control; yellow: 0.1 mg kg^{-1}; green: 1 mg kg^{-1}: orange: 5 mg kg^{-1}; blue: 10 mg kg^{-1}. LoQ, limit of quantification; MF, Macaca Fascicularis; PFU, plaque forming units.

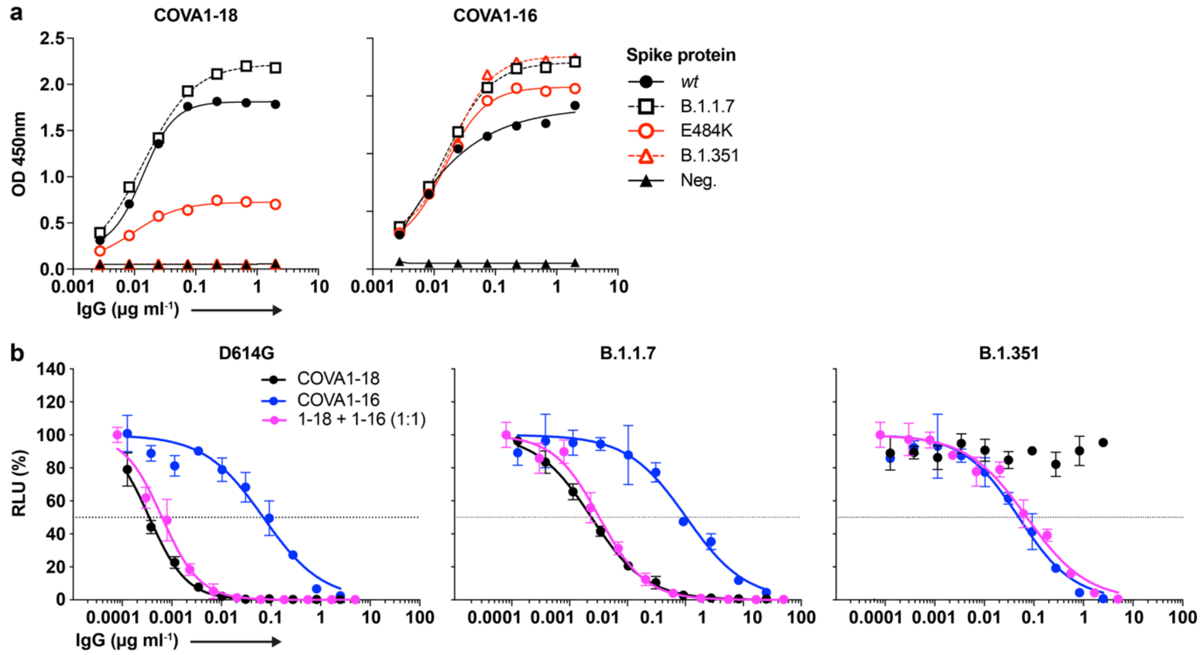

Supplementary Figure 6. Binding and neutralization activity of COVA1-18, COVA1-16 and a cocktail against variants. (**a**) ELISA binding curves of COVA1-18 and COVA1-16 against *wt* and several variant S proteins (representative of n ≥ 1 experiments). (**b**) Neutralization curves of COVA1-18, COVA1-16 and a 1:1 cocktail of both antibodies against pseudoviruses using D614G (left), B.1.1.7 (middle), and B.1.351 (right) strains (representative of n ≥ 2 experiments) (means ± SDs of 3 replicates). RLU, relative luminescence units.

Supplementary Table 1. Criteria for lung histopathology scoring in the hamster study. HPF, high power field (>10x); PMN, polymorphonuclear cells/heterophils; MNC, mononuclear cells including lymphocytes and macrophages; PVC, Peri-vascular cuff.

	**Score**	**0**	**1**	**2**	**3**	**4**
**A**	**Extent of inflammation (% tissue involved)**	0	<10	10-30	30-60	>60
**B**	**Inflammatory foci type**	No inflammation	Patchy inflammatory foci, few (<2)	Patchy inflammatory foci, many (>2)	Large inflammatory foci, few (<2)	Large inflammatory foci, many (>2)
**C**	**Inter-alveolar septa (IAS)**	Thin and delicate	Thickened in <10% HPF	Thickened in <30% HPF	Thickened in <60% HPF	Thickened in >60% HPF
**D**	**Air ways**	Clear; no cells	Few cells in air way	Moderate cells in air way	More cells in air way; Epithelial hyperplasia	Occlusion of air way/epithelial hyperplasia or desquamation
**E**	**Alveoli/ perivascular cuff/blood vessels/ pleuritis/cell types**	Clear; no inflammatory cells	Few cells/ Few PMN or MNC	Moderate cells/ PVC/ mild congestion/mild pleuritis/mostly MNC	More cells/PVC/ more congestion and pleuritis/ more MNC and PMN	Abundant cells/large PVC/severe congestion or pleuritis/mixed cells

*Adapted from Matute-Bello, G. Acute Lung Injury in Animals Study Group. An official American Thoracic Society workshop report: features and measurements of experimental acute lung injury in animals. *Am J Respir Cell Mol Biol.* **44**, 725-38 (2011).

Supplementary Table 2. Parameter estimates of the viral dynamic model. RSE: relative standard error

Parameters	Unit	Description	Fixed effect (RSE%)	Sd of random effect (RSE%)
β_N	ml per copies per day	Virion infectivity	2.14×10^{-4}	0.378
β_T	ml per copies per day		1.68×10^{-3}	
p_N	copies d^{-1}	Viral production	3.32×10^{4}	0.552 (99.3)
p_T	copies d^{-1}		1.12×10^{4}	
f		Scaling factor for the subgenomic RNA	6.98 (66.7)	1.53 (36)
EC_{50N}	μg ml^{-1}	Concentration required to block infectivity by 50%	2.2	0.366 (133)
EC_{50T}	μg ml^{-1}		0.053	
EC_{90N}	μg ml^{-1}	Concentration required to block infectivity by 90%	19.8	
EC_{90T}	μg ml^{-1}		0.48	
δ	d^{-1}	Baseline clearance rate of productively infected cells	1.88	0.172
ka	d^{-1}	Absorption rate	4.45	
k	d^{-1}	Elimination rate	0.0549 (13.3)	0.225 (44.5)
V	ml kg^{-1}	Volume of distribution	88.7 (6.56)	
D	mg kg^{-1}	Administered dose of COVA1-18	10	

Supplementary Table 3. Comparison of the in vitro and in vivo activity of monoclonal antibodies tested in prophylaxis experiments in NHPs.

ACE2, Angiotensin Convertase Enzyme 2; Conf., conformation; d.p.i., days post-infection; NA, not assessed; PFU, plaque forming unit; PrEP, pre-exposure prophylaxis; RBD, Receptor-binding domain; TCID50, fifty-percent tissue culture infective dose; URT, upper respiratory tract (nasopharyngeal and/or tracheal swabs).

			Neutralizing activity		PK/PD results in PrEP in NHP models of SARS-CoV-2 infection				
**Monoclonal antibody**	**Targeting domain**	**IGH IGL**	**IC_{50} against D614G virus (ng ml^{-1})**	**IC_{50} against B.1.351 virus (ng ml^{-1})**	**$t_{1/2}$ (days)**	**Dose (mg kg^{-1})**	**Challenge dose**	**Maximal reduction in URT median peak gRNA viral load over controls (log_{10} copies per ml)**	**Maximal reduction in URT median peak sgRNA viral load over controls (log_{10} copies per ml)**
**COVA1-18**	RBD	IGHV3-66 IGLV7-46	0.7	>1000	12	10	1x 10^6 PFU	2-4 (2 d.p.i.)	>2 (2 d.p.i.)
**REGN-10933**	RBD, overlaps ACE2 binding site	IGHV3-11 IGKV1-33	1-10 [37]	>1000 [37]	NA	25 + 25	1.05 x 10^6 PFU	2-3 (2 d.p.i.) [39]	>1.5 (2 d.p.i.) [39]
**REGN-10987**	RBD, no overlap with ACE2 binding site	IGHV3-30 IGLV2-14	1-10 [37]	1-10 [37]	NA				
**Ly-CoV555**	RBD, overlaps ACE2 binding site, up/down conf.	IGHV1-69 IGKV1-39	1-10 [37]	>1000 [37]	13[3]	1-50	1.1 x 10^5 PFU	0-3 (3 d.p.i.) [41]	0-3 (3 d.p.i.) [41]
**Ly-CoV016 (CB6)**	RBD, overlaps ACE2 binding site, up conf.	IGHV3-66 IGKV1-39	10-100 [37]	>1000 [37]	NA	50	1 x 10^5 TCID50	4-5 (4 d.p.i.) [42]	NA
**COV2-2196**	RBD, overlaps ACE2 binding site, up conf.	IGHV3-30 IGLV2-14	1-10 [37]	10-100 [37]	NA	50	1.1 x 10^4 PFU	NA	>5 (2 d.p.i.) [40]

Supplementary Table 4. Primers used for SARS-CoV-2 RT-PCR.

Name	Sequences (5'-3')	Length (bases)	PCR product size	Ref.
**RdRp gene / nCoV_IP4**				
nCoV_IP4-14059Fw	GGTAACTGGTATGATTTCG	19		
nCoV_IP4-14146Rv	CTGGTCAAGGTTAATATAGG	20	107 bp	1
nCoV_IP4-14084Probe(+)	TCATACAAACCACGCCAGG [5']Fam [3']BHQ-1	19		
**E gene / E_Sarbeco**				
E_Sarbeco_F1	ACAGGTACGTTAATAGTTAATAGCGT	18		
E_Sarbeco_R2	ATATTGCAGCAGTACGCACACA	20	125 bp	2
E_Sarbeco_P1	ACACTAGCCATCCTTACTGCGCTTCG [5']Fam [3']BHQ-1	20		

Supplementary Methods

Mouse experiment viruses

Ad5-hACE2 production. A seed stock for an E1/E3 deleted, non-replicating adenoviral vector based on human adenovirus type 5 (Ad5), encoding the human angiotensin converting enzyme-2 receptor (hACE2) under the control of a CMV promoter, was obtained from Iowa Viral Vector Core Facility. High titer stocks of Ad5-hACE2 were generated by amplification in T-Rex™-293 cells (Life Technologies, Carlsbad, CA), and purification with two rounds of cesium chloride (CsCl) ultracentrifugation. Virus was titered on T-Rex™-293, using the tissue culture infectious dose-50 (TCID50) endpoint dilution method with titers adjusted to PFU ml^{-1} as described previously[59,60].

SARS-CoV-2: Human isolate USA-WA1/2020 (BEI resources; NR-52281) was propagated in Vero E6 cells under BSL-3 containment in accordance with the biosafety protocols developed by the Icahn School of Medicine at Mount Sinai. Viral stocks were grown in Dulbecco Modified Eagle's Medium containing 2% fetal bovine serum, 2-[4-(2-hydroxyethyl)piperazin-1-yl]ethanesulfonic acid (HEPES), and penicillin–streptomycin for 72 h and were validated by genome sequencing. Cells were infected at a multiplicity of infection (MOI) of 0.1; mice were infected with 1×10^4 PFU.

Viral kinetic model (VK)

We used a previously published model characterizing nasopharyngeal and tracheal SARS-CoV-2 infection kinetics[36]. In this model, nasopharynx and trachea are modeled as two separated compartments described by a target cell limited model[61–63] as follows:

$$\frac{dT^x}{dt} = -\beta^x T^x V_I^x \quad (1)$$

$$\frac{dI_1^x}{dt} = \beta^x T^x V_I^x - kI_1^x \quad (2)$$

$$\frac{dI_2^x}{dt} = kI_1^x - \delta I_2^x \quad (3)$$

$$\frac{dV_I^x}{dt} = p^x \mu I_2^x - cV_I^x \quad (4)$$

$$\frac{dV_{NI}^x}{dt} = p^x(1-\mu)I_2^x - cV_{NI}^x \quad (5)$$

Where $T, I_1, I_2, V_I \; and \; V_{NI}$ are the densities of target cells, infected non-productive cell, infected productive cell, infectious virus and non-infectious virus respectively and the subscript x denotes the compartment of interest, either nasopharynx or trachea. Target cells (T) are infected at a rate β by infectious virus (V_I). Infected cells enter an eclipse phase (I_1) where they do not produce virions before becoming productively infected cells (I_2) at rate k. I_2 cells then produce virions at a rate p per days and are lost at a rate δ. A proportion μ of produced virions are infectious (V_I) and the remaining $(1-\mu)$ are non-infectious viruses (V_{NI}), both cleared at a rate c.

We assumed that subgenomic RNA, V_{sg}^x , is a proxy for the number of infected cells, and we wrote $V_{sg}^x = f(I_1^x + I_2^x)$ (6)

Where f is a scaling factor between sgRNA and the number of infected cells.

COVA1-18 pharmacokinetics model (PK)

We made use of a pharmacokinetic model with one compartment and a linear elimination to describe the dynamics of the COVA1-18 neutralizing antibody. The plasma concentration is given by the following analytical solution[64]:

$$C(t) = \frac{D}{V}\frac{ka}{ka-k}\left(e^{-k(t-t_d)} - e^{-ka(t-t_d)}\right) \quad (7)$$

Where $D, V, k, ka \; and \; t_d$ are the dose administered, the volume of distribution, the elimination rate, the absorption rate and the initiation of treatment, respectively. As the absorption phase could not be estimated, we fixed $k_a = 4.45 \, d^{-1}$, which corresponds to a T_{max} of 1 d, as observed in the data.

Concentration-Effect relationship (PK/PD)

To describe the interaction between the neutralizing antibody concentration and its efficacy, we used an E_{max} model, where the efficacy is linked to the antibody plasma concentration as:

$$\eta(t)^x = \frac{C(t)}{EC_{50}^x + C(t)} \quad (8)$$

Where EC_{50} is the plasma concentration of COVA1-18 required to reduce infectivity by 50%. Because the drug concentration, and hence the drug efficacy, is time-dependent, we calculated the mean individual efficacy over the first 10 days of infection as given by $\eta_{mean}^x = \int_{t=0}^{10} \eta(u)^x du$

Fixed parameters

We used the same assumptions as in [36].We fixed the $T_N(t = 0) = 1.25 \times 10^5$ and $T_T(t = 0) = 2.25 \times 10^4$ cells in nasopharynx and trachea respectively. Second, we assumed the proportion of infectious viruses μ remained constant over time and equal to 10^{-4}.Third, we fixed the eclipse phase duration to $k = 3\ day^{-1}$ and the viral clearance $c = 10\ d^{-1}$.

Parameter estimation and statistical methods

Parameter estimation was performed using maximum-likelihood methods using the stochastic approximation expectation-maximisation (SAEM) algorithm implemented in MONOLIX[65,66]. The PK/PD model was build using a two-stage approach. We fitted the PK data and injected the individual estimated parameters in the VK model. The estimated parameters for the PK models were V and k, and the estimated parameters for the VK model were $f, EC_{50}^T, EC_{50}^N, p^T\, and\ p^N$.

Simulation settings

The model was used to evaluate the effects of different experimental setting, such as the virus infectious dose, the timing of treatment initiation, or the dose of COVA1-18. Using the parameters given in Supplementary Table 2, we simulated different experimental designs and observed the predicted viral load (Genomic and Subgenomic) for both nasopharynx and trachea compartments. Several scenarios were assessed, according to inoculum size $(10^4, 10^5\ and\ 10^6\ PFU)$, COVA1-18 dose $(0.1, 1, 5, and\ 10\ mg\ per\ kg)$, and timing of treatment initiation $(-1\ d.p.i. +1\ d.p.i.)$. The simulations were performed using the Simulx package on R.3.6.0.
