# Supplementary material for: COVA1-18 neutralizing antibody protects against SARS-CoV-2 in three preclinical models
Source: Nat Commun. 2021 Oct 20;12:6097. doi: 10.1038/s41467-021-26354-0 (PMC8528857; doi:10.1038/s41467-021-26354-0)
Supplement: Supplementary file 3 — Reporting Summary [file 41467_2021_26354_MOESM3_ESM.pdf]

nature research

Corresponding author(s): Roger Le Grand, Marit J. van Gils, Rogier W. Sanders

Last updated by author(s): 21.09.2021

# Reporting Summary

Nature Research wishes to improve the reproducibility of the work that we publish. This form provides structure for consistency and transparency in reporting. For further information on Nature Research policies, see our Editorial Policies and the Editorial Policy Checklist.

## Statistics

For all statistical analyses, confirm that the following items are present in the figure legend, table legend, main text, or Methods section.

| n/a | Confirmed | |
|---|---|---|
| ☐ | ☒ | The exact sample size (*n*) for each experimental group/condition, given as a discrete number and unit of measurement |
| ☐ | ☒ | A statement on whether measurements were taken from distinct samples or whether the same sample was measured repeatedly |
| ☐ | ☒ | The statistical test(s) used AND whether they are one- or two-sided<br>*Only common tests should be described solely by name; describe more complex techniques in the Methods section.* |
| ☐ | ☒ | A description of all covariates tested |
| ☐ | ☒ | A description of any assumptions or corrections, such as tests of normality and adjustment for multiple comparisons |
| ☐ | ☒ | A full description of the statistical parameters including central tendency (e.g. means) or other basic estimates (e.g. regression coefficient) AND variation (e.g. standard deviation) or associated estimates of uncertainty (e.g. confidence intervals) |
| ☐ | ☒ | For null hypothesis testing, the test statistic (e.g. *F*, *t*, *r*) with confidence intervals, effect sizes, degrees of freedom and *P* value noted<br>*Give P values as exact values whenever suitable.* |
| ☒ | ☐ | For Bayesian analysis, information on the choice of priors and Markov chain Monte Carlo settings |
| ☒ | ☐ | For hierarchical and complex designs, identification of the appropriate level for tests and full reporting of outcomes |
| ☒ | ☐ | Estimates of effect sizes (e.g. Cohen's *d*, Pearson's *r*), indicating how they were calculated |

*Our web collection on statistics for biologists contains articles on many of the points above.*

## Software and code

Policy information about availability of computer code

| | |
|---|---|
| Data collection | Data was collected using classical Excel Files (Excel 2016) or collected on IntelliSpace Portal 8 (Philips Healthcare).<br>Macaque data were stored in a custom LIMS called BatLab.<br>BLI data were acquired with Octet Data Acquisition 10.0.03.12 (ForteBio). |
| Data analysis | Data was analyzed using GraphPad Prism v8 and Microsoft Excel.<br>Modeling was done using R and Monolix v2019. The code we used is available in Supplementary Information<br>BLI data were analyzed with Octet Analysis HT 10.0.3.7 (ForteBio).<br>Virus sequencing was performed on a GridION (Oxford Nanopore Technologies) for 72h with high-accuracy Guppy basecalling (v3.2.10). After sequencing, demultiplexing was performed using Guppy v4.0.14 with the option --require_barcodes_both_ends to ensure high quality demultiplexing. Reads were then filtered by Nanoplot v1.28.1 based on length and quality to select high quality reads. Then, reads were aligned on the SARS-CoV-2 reference genome NC_045512.2 using minimap2 v2.17. Primary alignments were filtered based on reads length alignment and reads identity. Reads were basecalled and demultiplexed with Guppy 4.0.14. The potential clonal and subclonal variants were detected with a custom pipeline based on ARTIC network workflow. Longshot v0.4.1 was used for variant detection. |

For manuscripts utilizing custom algorithms or software that are central to the research but not yet described in published literature, software must be made available to editors and reviewers. We strongly encourage code deposition in a community repository (e.g. GitHub). See the Nature Research guidelines for submitting code & software for further information.

## Data

Policy information about availability of data

All manuscripts must include a data availability statement. This statement should provide the following information, where applicable:

- Accession codes, unique identifiers, or web links for publicly available datasets
- A list of figures that have associated raw data
- A description of any restrictions on data availability

The viral sequencing data used in Supplementary Figure 3 have been deposited in the SRA repository under the accession code PRJNA758764 (PRJNA758764 - SRA - NCBI (nih.gov)). All the other raw data generated in this study are provided in the Supplementary Information Data files.

# Field-specific reporting

Please select the one below that is the best fit for your research. If you are not sure, read the appropriate sections before making your selection.

☒ Life sciences ☐ Behavioural & social sciences ☐ Ecological, evolutionary & environmental sciences

For a reference copy of the document with all sections, see nature.com/documents/nr-reporting-summary-flat.pdf

# Life sciences study design

All studies must disclose on these points even when the disclosure is negative.

| | |
|---|---|
| Sample size | Sample size was determined as the minimal number allowing non-parametric statistical analysis while complying with the 3Rs rule on reducing, replacing and refining the use of animals for scientific purpose. |
| Data exclusions | No data has been excluded from analysis. |
| Replication | Replicates were performed for all measurments within each assay (duplicates for Ni-NTA ELISAs and PCR, triplicates for ELISA and pseudovirus neutralization assays). |
| Randomization | Mice, golden Syrian hamsters and cynomolgus macaques were randomly assigned to control and treatment groups. For experiments that do not include animals, this is not relevant as they only include animal samples that were all tested at the same time. |
| Blinding | Animals care, clinical examination and sampling was not blinded because of constraints associated to BSL3/BSL4 containement.<br>Cynomolgus macaque viral loads and CT scoring were determined blindly.<br>Hamsters pathology scores were determined blindly.<br>Mice study was performed blindly in the sense that treatments were coded.<br>Other experiments such as ELISA and PCR were not blinded but included controls and standards and were performed in duplicates or triplicates. |

# Reporting for specific materials, systems and methods

We require information from authors about some types of materials, experimental systems and methods used in many studies. Here, indicate whether each material, system or method listed is relevant to your study. If you are not sure if a list item applies to your research, read the appropriate section before selecting a response.

### Materials & experimental systems

| n/a | Involved in the study | |
|---|---|---|
| ☐ | ☒ | Antibodies |
| ☐ | ☒ | Eukaryotic cell lines |
| ☒ | ☐ | Palaeontology and archaeology |
| ☐ | ☒ | Animals and other organisms |
| ☒ | ☐ | Human research participants |
| ☒ | ☐ | Clinical data |
| ☒ | ☐ | Dual use research of concern |

### Methods

| n/a | Involved in the study | |
|---|---|---|
| ☒ | ☐ | ChIP-seq |
| ☒ | ☐ | Flow cytometry |
| ☒ | ☐ | MRI-based neuroimaging |

## Antibodies

| | |
|---|---|
| Antibodies used | COVA1-18 (in house - Brouwer et al. 2020, 10.1126/science.abc5902)<br>Goat anti-human IgG-HRP (Jackson Immunoresearch): cat. # 109-035-003; RRID: AB_2337577<br>Goat anti-Human IgG H+L (monkey pre-adsorbed): Novus Biologicals, Cat# NB7487<br>Goat anti-Human IgG, Monkey ads-HRP: Southern Biotech, Cat# 2049-05<br>Goat Anti-Human IgG, Monkey ads-BIOT: Southern Biotech, Cat# 2049-08 |

Goat anti-Human IgG λ: Southern Biotech, Cat# 2070-01
Goat anti-Human IgG κ: Southern Biotech, Cat# 2060-01
Cynomolgus IgG standard, Molecular Innovations, Cat# CY-GF-10MG
Mouse anti-Monkey IgG BIOT, Southern Biotech, Cat# 4700-08
Monkey IgM, Molecular Innovations, Cat# MK-IGM-0.1MG
Anti-Monkey IgM (μ-chain specific)-Biotin antibody produced in goat; Sigma Aldrich; Cat# : SAB3700782
Anti-Mouse IgG-HRP: Abcam, Cat# ab6823
Monoclonal antibody cocktail composed of SARS-CoV-2 spike and SARS-CoV-2 nucleoprotein (Center for Therapeutic Antibody Discovery; NP1C7C7)

Validation

Except COVA1-18 (Brouwer et al. (2020), Science) and the monoclonal antibody cocktail provided by the center for therapeutic antibody discovery were commercially available .

For cynomolgus IgG standard, the manufacturer states it is specific to Cynomolgus macaques and has been tested for ELISA and WB (https://mol-innov.com/functions/admin/shipper/shipper.php?file_name=CY-GF&qty=1&no=HATIII&company=&submit=Submit!). This standard has been successfully used previously on an HIV antigen (https://doi.org/10.1016/j.omtn.2021.06.008).

For Monkey IgM standard, the manufacturer states : "Purified from normal rhesus monkey serum using size exclusion chromatography. >90% pure by SDS-PAGE. Major bands of ~70 and ~25 kDa indicative of the heavy and light chains of IgM on reducing SDS PAGE gels. IgG and IgA levels <2%"  and that it can be used for ELISA and WB (https://mol-innov.com/functions/admin/shipper/shipper.php?file_name=MK-IGM&qty=1&no=HATIII&company=&submit=Submit!)

# Eukaryotic cell lines

Policy information about cell lines

Cell line source(s)

African green monkey kidney VERO C1008 cell line (Vero 76, clone E6, Vero E6) obtained from ATCC (Cat# CRL-1586);
HEK 293T cells were obtained from ATCC (Cat# CRL-11268);
HEK 293T/ACE2 cells were obtained from Paul Bienasz (cf Schmidt et al. (2020), J. Exp. Med. 217, e20201181)
FreeStyle 293F cells were obtained from Thermo Fisher (Cat# R79007);
ExpiCHO-S cells were obtained from Thermo Fisher (Cat# A29127)

Authentication

These cell lines have been directly obtained from suppliers and not authenticated in-house.

Mycoplasma contamination

Confirmed negative test for mycoplasma contamination.

Commonly misidentified lines
(See ICLAC register)

No commonly misidentified cell lines were used in the study.

# Animals and other organisms

Policy information about studies involving animals; ARRIVE guidelines recommended for reporting animal research

Laboratory animals

The study have included 12 cynomolgus macaques (Macaca fascicularis) aged 3-6 years, both of male and female gender, 10 female golden Syrian hamsters aged 6-7 weeks and 13 female Balb/cJ mice aged 7 weeks.
All mice were housed in a temperature controlled environment with twelve hours of light per day at the Center for Comparative Medicine and Surgery (CCMS) at Icahn School of Medicine at Mount Sinai (New York, NY, USA). All experiments involving viral infections were carried out in a CDC/ USDA-approved BSL-3 facility at CCMS and animals were transferred into the facility four days prior to onset of experiments. Mice were housed in ventilated cages with ad libitum access to food and water.

Wild animals

No wild animals were used in the study.

Field-collected samples

No field collected samples were used in the study.

Ethics oversight

The protocol # 2004049 for the golden Syrian hamster experiment was approved by the Institutional Animal Care and Use Committee (IACUC) of the University of Texas Medical Branch at Galveston (UTMB).

The mouse experimental study was approved by the Icahn School of Medicine at Mount Sinai Institutional Animal Care and Use Committee (IACUC-2017-0170 and IACUC-2017-0330).

Male and female cynomolgus macaques (Macaca fascicularis), aged 3-6 years and originating from Mauritian AAALAC certified breeding centers were used in this study. All animals were housed in IDMIT infrastructure facilities (CEA, Fontenay-aux-roses), under BSL-2 and BSL-3 containment when necessary (Animal facility authorization #D92-032-02, Préfecture des Hauts de Seine, France) and in compliance with European Directive 2010/63/EU, the French regulations and the Standards for Human Care and Use of Laboratory Animals, of the Office for Laboratory Animal Welfare (OLAW, assurance number #A5826-01, US). The protocols were approved by the institutional ethical committee “Comité d’Ethique en Expérimentation Animale du Commissariat à l’Energie Atomique et aux Energies Alternatives” (CEtEA #44) under statement number A20-011. The study was authorized by the “Research, Innovation and Education Ministry” under registration number APAFIS#24434-2020030216532863.

Note that full information on the approval of the study protocol must also be provided in the manuscript.
